# Roles of orexinergic and noradrenergic neuronal activity in ketamine-induced sedation: a study using an orexin-ataxin-3 transgenic rat model

**DOI:** 10.1007/s00540-025-03521-x

**Published:** 2025-06-09

**Authors:** Mitsuru Tonosaki, Tetsuya Kushikata, Yoshikazu Nikaido, Daiki Takekawa, Hirotaka Kinoshita, Jyunichi Saito, Kazuyoshi Hirota

**Affiliations:** 1 Intensive Care Unit, Hirosaki University Hospital, Honcho 53, Hirosaki, Aomori 0368563 Japan; 2 Department of Anesthesiology, Hirosaki University Graduate School of Medicine, Zaifu 5, Hirosaki, Aomori 0368562 Japan; 3 Department of Metabolomics Innovation, Hirosaki University Graduate School of Medicine, Zaifu 5, Hirosaki, Aomori 0368562 Japan; 4 Department of Anesthesiology, Hirosaki University Hospital, Honcho 53, Hirosaki, Aomori 0368563 Japan; 5 Department of Perioperative Medicine for Community Healthcare, Hirosaki University Graduate School of Medicine, 5 Zaifu-cho, Hirosaki, 0368562 Japan; 6 Department of Perioperative Stress Management, Hirosaki University Graduate School of Medicine, 5 Zaifu-cho, Hirosaki, 036-8562 Japan

**Keywords:** Ketamine, Orexin, Brain noradrenergic neuron

## Abstract

**Purpose:**

To investigate the role of brain noradrenergic and orexinergic activity in ketamine-induced sedation.

**Methods:**

We used orexin neuron-deficient transgenic rats (orexin/ataxin-3) and wild-type controls. Noradrenaline and orexin levels were measured in the pons, hypothalamus, and cerebral cortex. Ketamine-induced loss-of-righting reflex (LORR) was assessed under modulation of noradrenergic or orexinergic activity.

**Results:**

Wild-type rats had higher noradrenaline and orexin levels than transgenic rats across all regions except hypothalamic noradrenaline. Noradrenaline and orexin were correlated in the pons and cortex. Transgenic rats had a shorter LORR duration than wild-type rats (36.3 ± 10.4 vs. 46.7 ± 5.2 min, P = 0.002). Noradrenergic activation via intraperitoneal yohimbine prolonged LORR in both genotypes (wild-type: 38.8 ± 4.9 vs. 71.9 ± 15.3 min at 3.3 mg/kg, P = 0.002; transgenic: 28.1 ± 3.9 vs. 71.9 ± 24.8 min, P < 0.001). Noradrenergic deactivation by DSP4 reduced LORR duration (wild-type: 43.3 ± 2.18 vs. 36.4 ± 6.0 min, P = 0.005). Intracerebroventricular orexin (1.0 nmol) shortened LORR (44.0 ± 16.7 vs. 30.1 ± 15.5 min, P = 0.001), but co-administration of selective orexin type 1 receptor antagonist YNT-1310 (100 nmol) counteracted this effect. Notably, orexin or DSP4 reduced LORR duration in wild-type rats but prolonged it in transgenic rats (e.g., wild-type: 40.8 ± 6.2 vs. 32.5 ± 5.3 min with orexin, P = 0.0001; transgenic 28.6 ± 6.2 vs. 42.1 ± 5.6 min, P = 0.0026).

**Conclusion:**

Orexin-preserved noradrenergic activity supports the typical ketamine-induced sedation profile, highlighting their interactive role in modulating anesthetic depth.

## Introduction

Endogenous sleep circuits are thought to be involved in the mechanism of the loss of consciousness by general anesthesia [1–4]. Orexin (OX) is an endogenous wake-promoting substance [5], and it facilitates emergence from various anesthetics; barbiturates [6], propofol [7, 8], and isoflurane [9].

A previous study [10] examined the postsynaptic effects of exogenous OX administration on anesthetic status. However, the role of OXergic neuronal activity in the sedative effects of ketamine remains unclear. In this study, we used transgenic (TG) rats with genetically ablated OX-producing neurons [11] as a model of OXergic neuronal inactivation.

Physiologically, OX activates locus coeruleus (LC) noradrenaline (NA) neurons via type 1 OX receptors [12]. Therefore, the TG rats used in this study may exhibit altered LC–NA activity compared to wild-type (WT) rats. LC–NA activity influences ketamine-induced sedation; ketamine prolongs LC–NA activity by approximately 400% above baseline [10], whereas selective destruction of LC–NA neurons attenuates its sedative effects [13]. These findings suggest that the interaction between OX and LC–NA may be a critical component of ketamine sedation.

In this study, we quantified OX and NA content in untreated TG and WT rats as indicators of OXergic and NAergic neuronal activity. Measurements were taken in the pons (containing LC–NA cell bodies), hypothalamus (containing OX-producing neurons), and cerebral cortex (receiving projections from LC–NA neurons). Neuronal activation and inactivation in these brain regions are known to modulate wakefulness, sleep, and anesthesia [14].

We also examined correlations between NA and OX contents in each region. Moreover, we investigated how noradrenergic and OXergic activity influences ketamine sedation by administering yohimbine (an α2 receptor antagonist), DSP4 (a selective LC–NA neurotoxin), and YNT-1310 (a selective type 1 OX receptor antagonist) to TG and WT rats. This series of experiments aimed to clarify the role of OXergic and NAergic neuronal activity in modulating the sedative effects of ketamine.

## Methods

With the approval of the Institutional Committee on Animal Research of Hirosaki University Graduate School of Medicine and in accordance with the ARRIVE guidelines, we used male OX/ataxin-3 TG rats (weight: 388.7 ± 48.5 g, n = 113) and corresponding WT rats (weight: 401.1 ± 56.2 g, n = 141).

The OX/ataxin-3 TG rats, expressing a truncated Machado–Joseph disease gene product (ataxin-3) with an expanded polyglutamine stretch specifically in hypocretin neurons, were gifted by Prof. Masashi Yanagisawa (International Institute for Integrative Sleep Medicine, University of Tsukuba, Japan). The TG rats express truncated human Ataxin-3 with 77 polyglutamine repeats in OX-expressing cells [11, 15]. By 4 weeks of age, the number of OX-expressing cells was notably diminished in the lateral hypothalamus of OX/Ataxin-3 TG rats as a result of polyglutamine toxicity [11]. The TG line was maintained as homozygotes, and PCR confirmed their homogeneity with tail DNA according to a previous study [11]. TG rats were cross-bred with the WT rats to create littermates that were either hemizygous (one copy of OX/ataxin-3) or WT. All experiments used male TG rats aged 8–16 weeks and WT rats aged 7–16 weeks. The TG and WT littermates were group-housed at 24.0 ± 2.0 °C under a 12-h light/dark cycle (lights on at 8:00 am), and food and water were available freely except on the experiment day. All experiments were carried out following the ARRIVE Guideline and the guidelines for animal research issued by the Hirosaki University Graduate School of Medicine. This study was approved by the Animal Research Committee of Hirosaki University (approval number AE01-2023-123). Each experiment was performed between 12:00 and 16:00 to control the diurnal rhythm of sleep–wakefulness.

## Experimental protocol

### Measurement of the NA and OX content in the brain

Brains from ketamine-untreated WT and TG rats were collected for NA and OX content analysis (TG: n = 10; WT: n = 10). All rats were decapitated, and their brains were rapidly removed. The pons, hypothalamus, and cerebral cortex were dissected from the surrounding structures, weighted, and then immediately sonicated in physiological saline. The supernatant was collected after centrifugation of the sonicated material stored at − 70 °C until the following assays. Commercially available OX enzyme-linked immunosorbent assay kits were used to quantify OX levels (Peninsula Laboratories International, San Carlos, CA, USA). The mean intra and inter-assay coefficients of variation were 5%. NA levels were determined by the high-performance liquid chromatographical assay (ESA Coulochem Model 5100A, Tokyo, Japan) using a C18 reverse-phase column (4.6 × l50 mm, MC Medical, Tokyo, Japan) with a mobile phase consisting of buffer (0.05 M NaH_2_PO_4_, 0.05 M CCl_3_COOH, 0.7 mM CH_3_(CH_2_)11OSO_3_Na, 0.02 mM EDTA2Na, pH 3.4 at 40℃): acetonitrile: methanol in the ratio of 85:10:5 (v/v/v) with a flow rate of 1 mL/min. NA was detected using an electrochemical detector at 300 mV (optimum voltage for oxidation). The lower detection limit was 15 pg/mL, and the intra-assay coefficient of variation was 3.3%.

## Loss-of-righting assay

The rats (TG and WT) received 100 mg/kg ketamine (Daiichi Sankyo Co. Ltd, Tokyo, Japan) intraperitoneal injection as described previously [16]. The induction time was defined as the duration from the ketamine injection to loss-of-righting. Loss-of-righting duration was defined as the time from the loss-of-righting to recovery to perform three-successive-righting [13, 17]. We conducted five patterns of the loss-of-righting assay. In the first pattern, 30 rats (TG and WT; n = 15 each) received ketamine alone. In the second pattern, TG and WT rats received the α2-adrenoceptor antagonist yohimbine at doses of 0.0 (vehicle), 1.0, 3.3, or 10.0 mg/kg (n = 8 per dose) intraperitoneally, 60 min prior to ketamine administration. In the third pattern, TG and WT rats (n = 12 per dose) received either 0 or 50 mg/kg N-(2-chloroethyl)-N-ethyl-2-bromobenzylamine hydrochloride (DSP4; Sigma–Aldrich, St. Louis, USA) intraperitoneally. After a 10-day recovery period, these rats received ketamine. In the fourth pattern, only WT rats (n = 7 per dose) received 100 nmol YNT-1310 (YNT-1310 Disulfate Hydrate; FUJIFILM Wako Chemical Co., Osaka, Japan), a selective OX type 1 receptor antagonist, with or without 1.0 nmol OX (Orexin A trifluoroacetate salt; American Peptide Sunnyvale, CA, USA) administered via intracerebroventricular (icv) injection, followed by ketamine 60 min later. In the fifth pattern, TG and WT rats (n = 8 per dose) received 0 or 50 mg/kg DSP4 intraperitoneally. After a 10-day recovery period, these rats received 0.0 or 1.0 nmol OX via icv injection, followed by ketamine administration 60 min later.

## Statistical analysis

Based on our previous studies, the required sample sizes to achieve a β power of 0.8 with an α error probability of 0.05 (G*Power 3.1.9.7) [18] were as follows: 4 for comparison of ketamine-induced loss-of-righting reflex duration between WT and TG rats (effect size = 3.33) [10]; 9 for multiple comparisons of ketamine and OX interaction (effect size = 1.47) [10]; 18 for multiple comparisons of ketamine and NA interaction (effect size = 0.89) [13], 14 for comparison of brain NA content between WT and TG rats (effect size = 1.69) [19]; and 8 for comparison of brain OX content between WT and TG rats (effect size = 2.99) [19]. The actual sample sizes used were larger than required to allow for a greater margin of error. The data are expressed as the mean ± standard deviation. Statistical analyses were performed using the unpaired t-test to compare the duration of ketamine-induced loss-of-righting between WT and TG rats, OX and NA concentrations (pg/mg wet tissue), and body weight. Two-way ANOVA followed by Tukey’s multiple comparisons test was used to assess the effects of yohimbine and OX with or without DSP4 on ketamine-induced loss-of-righting duration. One-way ANOVA followed by Šídák’s multiple comparisons test was used to evaluate the effects of DSP4 and OX with or without YNT-1310 on ketamine-induced loss-of-righting duration. All statistical analyses were performed using GraphPad Prism version 9.1.2 for Windows (GraphPad Software, La Jolla, CA, USA). Statistical significance was set at P < 0.05. Pearson’s correlation analysis was also conducted to evaluate the relationship between brain OX levels and corresponding regional NA contents.

## Results

**Fig. 1:**
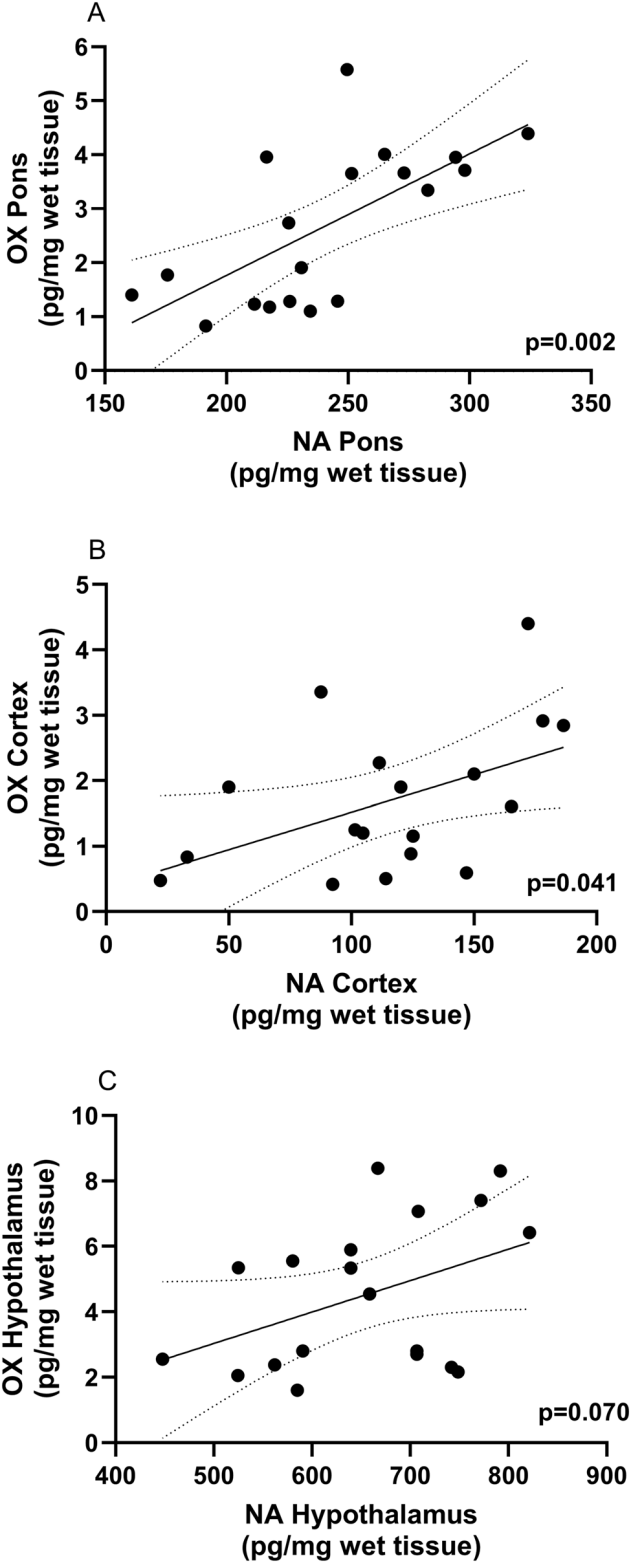
Noradrenaline levels were positively correlated with orexin levels in the rat pons and cerebral cortex. Pearson’s correlation analysis revealed significant relationships between noradrenaline and orexin in the pons (**A**) and cerebral cortex (**B**) but not in the hypothalamus (**C**). The neurotransmitter concentration data of orexin/ataxin-3 transgenic (n = 10) and wild-type (n = 10) rats were pooled and analyzed. The coefficients are presented with (95% confidence interval)

**Fig. 2:**
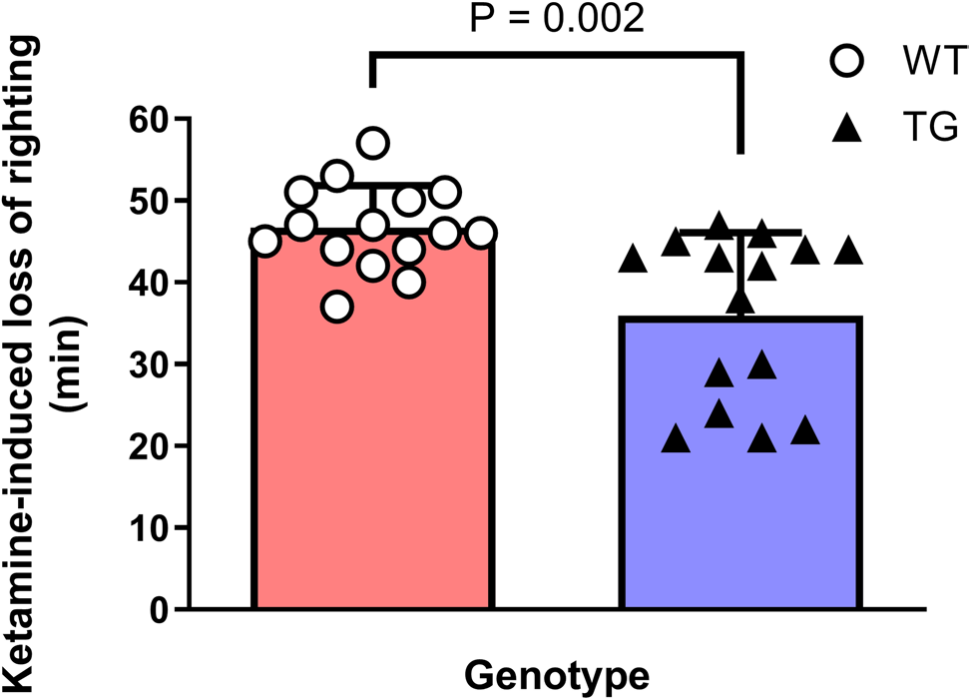
Orexin deficiency attenuated ketamine-induced loss-of-righting duration. Transgenic (TG; n = 15) rats displayed significantly shorter duration of loss-of-righting than wild-type (WT; n = 15) rats following 100 mg/kg ketamine intraperitoneal injection. The data are presented as the mean ± standard deviation. The red closed rectangle denotes the duration of ketamine-induced loss-of-righting in WT rats, whereas the blue closed rectangle denotes the duration in TG rats

We confirmed that in ketamine un-administered (control) rats, TG rats had reduced OX contents in the pons, hypothalamus, and cerebral cortex compared to WT rats (Table 1). NA concentrations in the pons and cerebral cortex—but not in the hypothalamus—were significantly lower in TG rats than in WT rats. To evaluate the interaction between NA and OX, we performed Pearson’s correlation analysis in each brain region (Fig. 1). Significant correlations were observed between OX and NA contents in the pons (Fig. 1A; r^2^ = 0.44, P = 0.002) and the cerebral cortex (Fig. 1B; r^2^ = 0.24, P = 0.041), but not in the hypothalamus (Fig. 1C; r^2^ = 0.18, P = 0.070). WT and TG groups displayed loss-of-righting induced by 100 mg/kg ketamine intraperitoneal injection (Fig. 2). The duration of ketamine-induced loss-of-righting was 46.7 ± 5.2 min in WT rats and was shortened to 35.9 ± 10.2 min in TG rats (t = 3.865, df = 28, P = 0.016).

**Table 1:** Inter-group comparison of noradrenaline and orexin in ketamine un-administrated wild and transgenic rats

Regions	Genotype				P value
	Wild	n	Transgenic	n	
Noradrenaline content (pg/mg wet tissue)					
Pons	268.1 ± 33.4	10	210.5 ± 28.6	9	0.0009
Cerebral cortex	138.7 ± 37.0	9	93.1 ± 47.7	9	0.0376
Hypothalamus	680.4 ± 94.0	10	617.8 ± 102.1	10	0.1706
Orexin content (pg/mg wet tissue)					
Pons	3.9 ± 0.7	10	1.3 ± 0.3	9	< 0.0001
Cerebral cortex	2.4 ± 1.1	9	1.0 ± 0.6	9	0.0033
Hypothalamus	6.4 ± 1.3	10	2.8 ± 0.4	9	< 0.0001

These comparisons were performed in ketamine un-administrated rats. We measured the noradrenaline and orexin levels without ketamine (ketamine un-administrated). The wild-type rats had higher noradrenaline and orexin contents than transgenic rats in all three regions, except for hypothalamic noradrenaline. Some data on ketamine-un-administrated were missing because of a technical error.

**Fig. 3:**
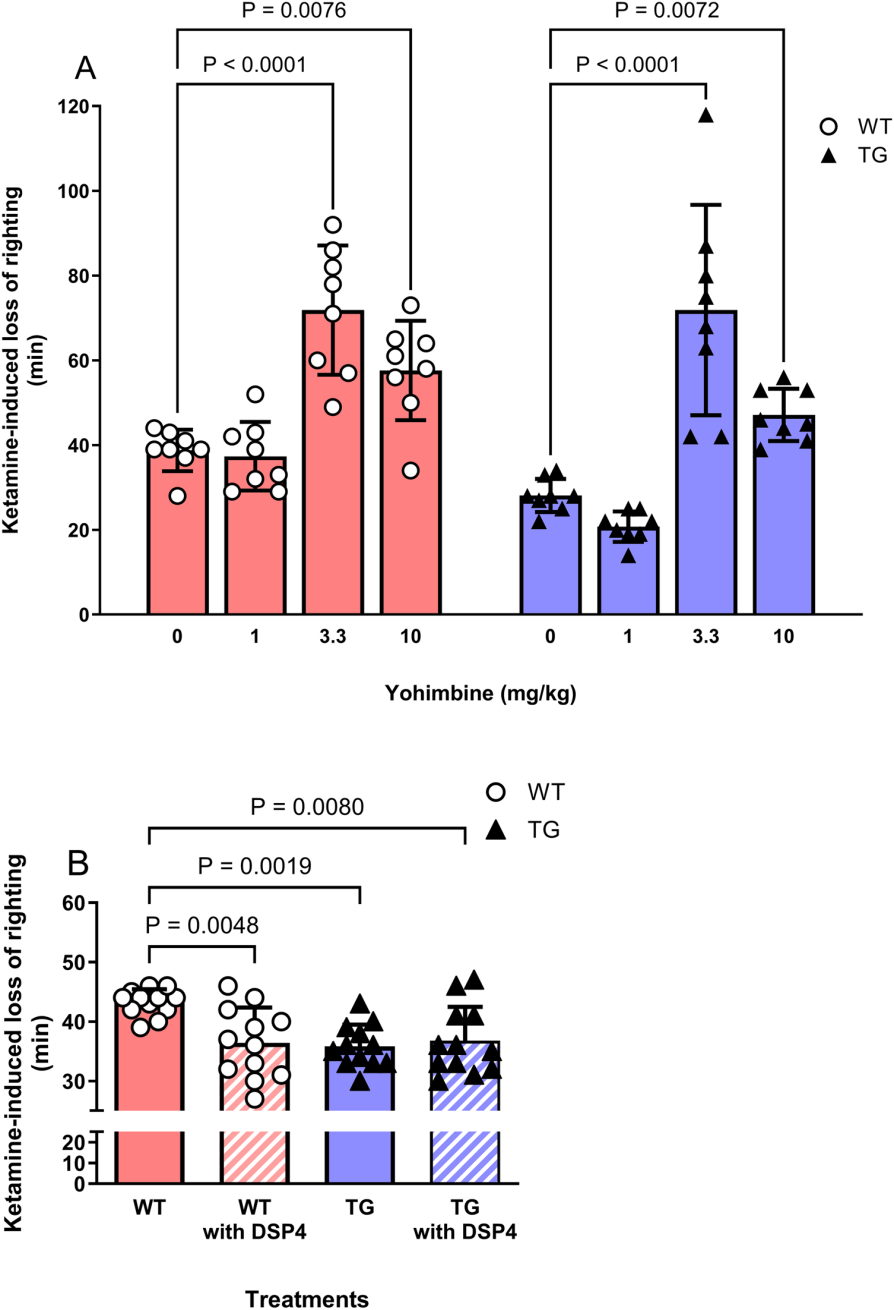
Central noradrenergic neuron activation was prolonged, whereas its inhibition shortened ketamine-induced loss-of-righting duration in wild-type (WT) and transgenic (TG) rats. WT (n = 8) and TG (n = 8) rats intraperitoneally pretreated with yohimbine (3.3 mg/kg and 10 mg/kg), alpha2-receptor antagonist, at doses showed longer loss-of-righting duration produced by 100 mg/kg ketamine administration than animals pretreated with saline (yohimbine 0 mg/kg; **A**). Conversely, DSP4, a selective toxin for locus coeruleus noradrenergic neurons, reduced ketamine-induced loss-of-righting duration in both genotypes (**B**; n = 8 each). The data are presented as the mean ± standard deviation. In **A**, the red closed rectangle denotes the duration of ketamine-induced loss-of-righting duration in WT rats, whereas the blue closed rectangle denotes the duration in TG rats. In **B**, the red and blue closed rectangles denote the duration of ketamine-induced loss-of-righting in WT (red) and TG (blue) without DSP4 pretreatment, and the hatched rectangles denote the duration of ketamine-induced loss-of-righting in WT (red) and TG (blue) with DSP4 pretreatment

Intraperitoneal injection of 3.3 mg/kg and 10.0 mg/kg doses of yohimbine (Fig. 3A), an α2-adrenoceptor antagonist, prolonged ketamine-induced loss-of-righting duration in both genotypes (two-way analysis of variance [ANOVA]: dose, F_3,56_ = 43.06, P < 0.0001; genotype, F_1,56_ = 9.98, P = 0.0026; dose × genotype, F_3,56_ = 1.34, P = 27; post-hoc comparisons: WT 0 mg/kg, 38.8 ± 4.9 min vs. 3.3 mg/kg, 71.9 ± 15.3 min, P = 0.002; vs. 10.0 mg/kg, 57.6 ± 11.7 min, P = 0.007; TG 0 mg/kg, 28.1 ± 3.9 min vs. 3.3 mg/kg, 71.9 ± 24.8 min, P < 0.001; vs. 10.0 mg/kg, 47.1 ± 6.2 min, P = 0.001). DSP4 (Fig. 3B) reduced ketamine-induced loss-of-righting duration in both genotypes (one-way ANOVA: dose, F_3,44_ = 6.73, P = 0.001; post-hoc comparisons: WT 0 mg/kg, 43.3 ± 2.18 min vs. WT 50 mg/kg, 36.4 ± 6.0 min, P = 0.005; vs. TG 0 mg/kg, 35.8 ± 3.6 min, P = 0.002; vs. TG 50 mg/kg, 36.8 ± 5.7 min, P = 0.008).

**Fig. 4:**
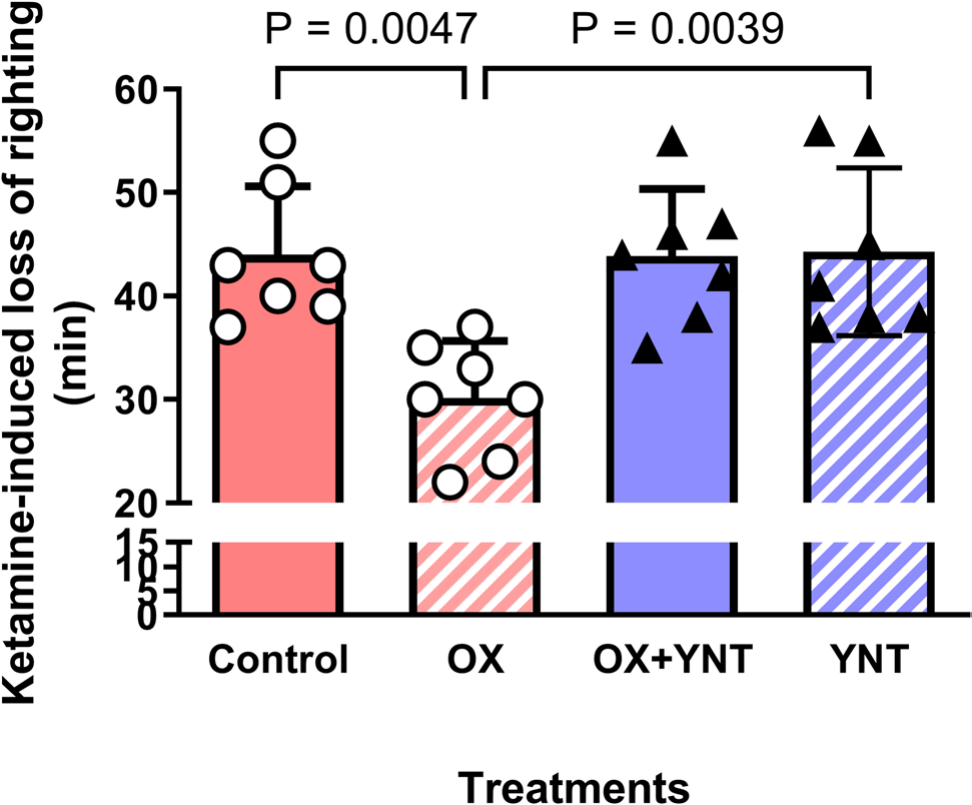
A selective orexin (OX) type 1 receptor antagonist, YNT-1310, counteracted the effect of OX on ketamine-induced loss-of-righting duration in wild-type rats. OX 1.0 nmol intracerebroventricular (icv) injection decreased ketamine-induced loss-of-righting duration, whereas YNT-1310 (100 nmol), a selective OX type 1 receptor antagonist, with or without OX 1.0 nmol icv, counteracted the effect of orexin on ketamine-induced loss-of-righting duration. The red closed rectangle denotes ketamine-induced loss-of-righting duration with physiological saline icv (control). The red hatched rectangle denotes ketamine-induced loss-of-righting duration with OX 1.0 nmol icv. The blue closed rectangle denotes ketamine-induced loss-of-righting duration with OX combined with YNT-1310 icv, whereas the blue hatched rectangle denotes ketamine-induced loss-of-righting duration with YNT-1310 alone icv

OX 1.0 nmol icv injection shortened ketamine-induced loss-of-righting duration (one-way ANOVA: F = 7.42, P = 0.001; OX 0.0 nmol, 44.0 ± 16.7 min vs. OX 1.0 nmol, 30.1 ± 15.5 min, P = 0.001); whereas YNT-1310 100 nmol with or without OX 1.0 nmol icv counteracted the effect of OX on the ketamine-induced loss-of-righting duration (Fig. 4).

**Fig. 5:**
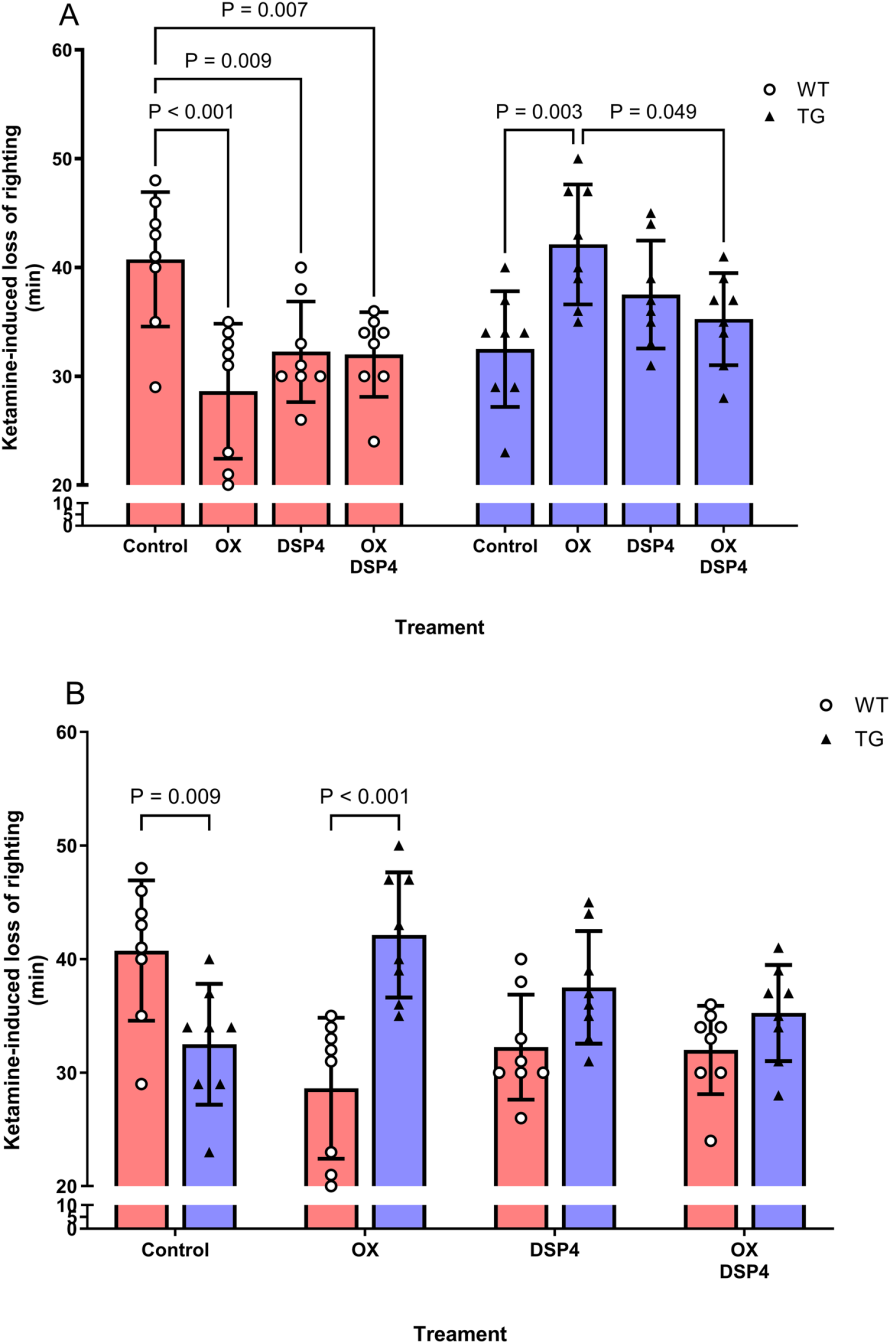
The effect of orexin (OX) on ketamine-induced loss-of-righting duration depends on central noradrenergic activity. OX 1.0 nmol icv or DSP4, a selective toxin for locus coeruleus noradrenergic neurons, decreased ketamine-induced loss-of-righting duration in WT rats, whereas in TG rats, the OX dose increased the duration (**A**). Ketamine induced a longer loss-of-righting duration in untreated WT rats than in untreated TG rats. OX 1.0 nmol icv prolonged ketamine-induced loss-of-righting duration in TG rats, bringing the duration closer to that of untreated WT rats. Noradrenergic inhibition by DSP4 abolished the OX effect on ketamine-induced loss-of-righting duration (**B**). The red closed rectangle denotes ketamine-induced loss-of-righting duration in WT rats. The blue closed rectangle denotes ketamine-induced loss-of-righting duration in TG rats

OX 1.0 nmol icv or DSP4 shortened ketamine-induced loss-of-righting duration in WT rats (two-way ANOVA: interaction, F_3,56_ = 12.00, P < 0.0001, genotype, F_1,56_ = 7.05, P = 0.010; post-hoc comparisons: WT OX 0.0 nmol, 40.8 ± 6.2 min vs. OX 1.0 nmol, 28.6 ± 6.1 min, P < 0.001; vs. DSP4 alone, 32.3 ± 4.6 min, P = 0.009; vs. OX 1.0 nmol + DSP4, 32.0 ± 3.9 min, P = 0.007). Conversely, in TG rats, OX prolonged the duration (two-way ANOVA: interaction, F_3,56_ = 12.00, P < 0.001; genotype, F_1,56_ = 7.05, P = 0.010; post-hoc comparisons: TG OX 0.0 nmol, 32.5 ± 5.3 min vs. OX 1.0 nmol, 42.1 ± 5.5 min, P = 0.013) and the duration was similar to that of untreated WT rats, with no significant difference (two-way ANOVA: interaction, F_3,56_ = 12.00, P < 0.001, genotype, F_1,56_ = 7.05, P > 0.999; post-hoc comparisons: WT OX 0.0 nmol, 40.8 ± 6.2 min vs. TG OX 1.0 nmol, 42.1 ± 5.5 min, P > 0.999) (Fig. 5A). Untreated WT rats showed a longer ketamine-induced loss-of-righting duration than untreated TG rats (two-way ANOVA: interaction, F_3,56_ = 12.00, P < 0.0001; genotype, F_1,56_ = 7.05, P = 0.010; post-hoc comparisons: WT OX 0.0 nmol, 40.8 ± 6.2 min vs. TG OX 0.0 nmol, 32.5 ± 5.4 min, P = 0.009). This effect was reversed by OX 1.0 nmol icv (post-hoc comparisons: WT OX 1.0 nmol, 28.6 ± 6.1 min vs. TG OX 1.0 nmol, 42.1 ± 5.5 min, P < 0.001). OX 1.0 nmol icv prolonged the duration in TG rats to a level similar to that of untreated WT rats. Noradrenergic inhibition by DSP4 abolished the effect of OX on ketamine-induced loss-of-righting duration (Fig. 5B).

## Discussion

Consistent with a previous study [11], we confirmed that the OX levels were reduced in the untreated TG rat brains. We also found that NA content was lower in the pons and cerebral cortex of TG rats. NA and OX levels were correlated in these regions, where LC noradrenergic neurons are innervated, but not in the hypothalamus, where non-LC noradrenergic neurons are present. In TG rats, the sedative effect of ketamine was reduced compared to WT. Ketamine-induced loss-of-righting duration changed with central noradrenergic activity, regardless of OX activity. When NA activity was prolonged with yohimbine, the ketamine-induced loss-of-righting duration was prolonged in WT and TG rats. Conversely, when NA activity was suppressed with DSP4, the duration was reduced in WT and TG rats compared to untreated WT. Meanwhile, orexinergic activity affected ketamine-induced loss-of-righting duration depending on central noradrenergic activity. In untreated WT rats with normal central noradrenergic activity, OX activation shortened the ketamine-induced loss-of-righting duration. Conversely, in TG rats with suppressed central noradrenergic activity, OX activation prolonged the duration, approaching the value observed in untreated WT rats.

NA content was also low in TG rats with reduced OX activity. As described below, OXergic neurons interact with NAergic neurons; thus, NA content in TG rats could be lower in parallel with OX levels. Noradrenergic LC neurons in the pons and OXergic neurons in the lateral hypothalamus reciprocally innervate each other, and inhibition of OXergic input attenuates LC neuronal activity [20]. The LC expresses OX type 1 receptors [21], and OX activates the LC via these receptors. The LC contains NA cell bodies; therefore, in TG rats with reduced OX activity, LC–NA activity may also be reduced, explaining the lower NA content in the pons. NA content was also reduced in the cerebral cortex, which receives noradrenergic projections only from the LC. In contrast, no difference in NA content was observed between TG and WT rats in the hypothalamus, which receives noradrenergic input from non-LC neurons. This result is consistent with previous findings that OX activates LC–NA [12]. In addition, NA content correlated with OX content in the pons and cerebral cortex but not in the hypothalamus, further supporting the interaction between OX and LC–NA neurons.

Previously, we reported that OX activation attenuated ketamine-induced loss-of-righting duration [10]. Based on this result, we predicted that ketamine-induced sedation would be enhanced in TG rats with low OX activity. However, contrary to our prediction, ketamine-induced loss-of-righting duration was attenuated in TG rats. One possibility is that the reduction in NA activity in TG rats may have weakened the sedative effects of ketamine, as the activation of central NA is required to exert the sedative effects of ketamine [10, 13, 22, 23]. This study demonstrated a reduction in NA levels in the TG rat brain. Activation of NA with yohimbine prolonged the duration of ketamine-induced sedation in TG and WT rats. Conversely, in rats with suppressed noradrenergic neurons induced by DSP4, the sedative effects of ketamine were reduced in WT and TG rats. These results suggest that NA activity influences the duration of ketamine-induced sedation more than OX activity. Therefore, lower brain NA levels in the TG rats could cause a shorter duration of ketamine-induced sedation.

In contrast, regarding the relationship between OX and the mechanism of ketamine sedation, the effects of OX icv were opposite in WT and TG rats. The difference in NA activity between the two groups could explain the differential OX effect. In TG rats with suppressed NAergic activity, OX icv may restore remaining noradrenergic neuronal activity to levels closer to that of WT rats; thus, the ketamine-induced loss-of-righting duration could resemble that of untreated WT rats. These results suggest that the role of OX in the mechanism of ketamine sedation depends on NAergic activity. Indeed, DSP4-induced LC–NA inhibition attenuated the effect of OX on ketamine-induced loss-of-righting duration in WT and TG rats.

We could not exclude any OX roles on the action of ketamine by interacting with other neurotransmitters such as acetylcholine, histamine, serotonin, and gamma-aminobutyric acid (GABA) [14, 24], which are involved in sedation and analgesia [2]. The TG rats differed from normal WT rats in various ways, including sleep–wake patterns [11]. These differences might affect the ketamine-induced sedation. Although we focused on the relationship with NA in the present study, other parts of the relationship between NA and ketamine should be further evaluated. Chemogenetic and optogenetic studies will overcome these limitations. It recommends studying male and female data to reject gender bias and obtain more universal findings. When obtaining data on females, it is always necessary to consider the influence of the estrous cycle. The estrous cycle changes OX dynamics [25], anesthesia [2], and analgesia [26]; thus, it is necessary to align female data with their estrous cycle. The estrous cycle of rodents is 3 to 4 days; however, obtaining data with the same estrous cycle at our facility was challenging. Therefore, although the data were limited to males, we reported the findings of sedation and analgesia induced by OX, NA, and ketamine. The data from females should be studied in future studies.

In conclusion, ketamine-induced sedation was altered by NAergic neuronal activity, regardless of OX neuronal activity. OX-preserved noradrenergic neuronal activity supported the typical ketamine-induced loss-of-righting response.

## Data Availability

The datasets used during the current study are available from the corresponding author on reasonable request.
